# Prospective study of dietary changes in cancer survivors for five years including pre- and post- diagnosis compared with those in cancer-free participants

**DOI:** 10.1038/s41598-023-27820-z

**Published:** 2023-01-18

**Authors:** Yuri Ishii, Ribeka Takachi, Junko Ishihara, Taiki Yamaji, Motoki Iwasaki, Manami Inoue, Shoichiro Tsugane, Norie Sawada

**Affiliations:** 1 Division of Cohort Research, National Cancer Center Institute for Cancer Control, 5-1-1 Tsukiji, Chuo-Ku, Tokyo, 104-0045 Japan; 2 Department of Food Science and Nutrition, Nara Women’s University Graduate School of Humanities and Sciences, Kitauoyahigashimachi Nara-Shi, Nara, 630-8506 Japan; 3 Department of Food and Life Science, Azabu University, 1-17-71 Fuchinobe, Chuo-Ku, Sagamihara-City, Kanagawa 252-5201 Japan; 4 Division of Epidemiology, National Cancer Center Institute for Cancer Control, 5-1-1 Tsukiji, Chuo-Ku, Tokyo, 104-0045 Japan; 5 Division of Prevention, National Cancer Center Institute for Cancer Control, 5-1-1 Tsukiji, Chuo-Ku, Tokyo, 104-0045 Japan; 6 National Institute of Health and Nutrition, National Institutes of Biomedical Innovation, Health and Nutrition, 1-23-1 Toyama, Shinjuku-Ku, Tokyo, 162-8636 Japan

**Keywords:** Nutrition, Public health, Epidemiology

## Abstract

The number of long-term survivors after a cancer diagnosis is increasing. Few investigations have compared survivors’ diets to their original pre-diagnosis dietary pattern or with the patterns of cancer-free controls. We examined the dietary changes in survivors for five years (i.e. before to after diagnosis) in cancer survivors, comparing them with cancer-free controls in a prospective cohort study in Japan. Using 1995–1998 for the baseline and 2000–2003 for the follow-up survey, a validated food frequency questionnaire was administered to 33,643 men and 39,549 women aged 45–74 years. During the follow-up period, 886 men and 646 women had developed cancer. Participants that had not been diagnosed with cancer served as controls. There was a greater decrease in the calorie intake (median change: − 168 kcal**/**d [Interquartile range: − 640, 278]) in male cancer survivors compared to controls (− 33 kcal**/**d [− 453, 380], *P* < .001). On comparison with cancer-free controls, multiple linear regression analysis revealed a significantly larger reduction in energy-adjusted ethanol intake for male cancer survivors (β =  − 0.36). There was no difference in changes in fruit and vegetable or red meat intake and no other significant differences in dietary changes between survivors and controls for either gender. This suggests that most dietary changes in survivors after cancer diagnosis are not systematically different from those that occur in people without a cancer diagnosis.

## Introduction

The age-standardized incidence rate of cancers has entered a phase of decline globally^1^. However, the number of cancer patients has been increasing^2^ in tandem with the increasing percentage of aged individuals in the population^3^. The survival rates of cancers are increasing^4^, suggesting that the number of long-term survivors after diagnosis will continue to increase. Thus, guidelines^5,6^ and accurate data on diet and lifestyles after diagnosis in association with prognosis and recurrence are increasingly important for public health management in survivors.

Persons diagnosed with cancer may try to improve their diet in accordance with recommendations they receive at the time of diagnosis. However, there is little evidence on whether consequent changes are lost or retained over time, compared to those who are not diagnosed with cancer. Some studies of dietary changes in cancer survivors have been retrospective^7–10^. Several studies have examined these changes prospectively, but among survivors only^11–16^. To our knowledge, few prospective studies have examined changes in dietary patterns among survivors compared with changes over time among cancer-free participants^17,18^, one of which examined changes in specific elements, such as fruit and vegetable intake^18^. Both were conducted in Western countries, Norway^17^ and France^18^, and no data have been reported for Asian populations. Asian populations tend to differ from Western populations with regard to the proportions of site-specific cancers (higher incidence of gastric and lower incidence of breast cancers)^2^ and dietary pattern (such as consumption of higher proportions of rice and lower proportions of red meat)^19^.

Here, we examined changes in consumption of food groups over five years in a large-scale prospective cohort study in Japan. Food groups were used as a suitable means of expressing the results of changes in dietary behavior. Elements of change in diet, including ethanol, were selected from The Third Expert Report^6^ of the WCRF/AIRC as having strong evidence (convincing or probable) and a suggestive causal relationship for primary prevention of breast, colorectal, and gastric cancer, the latter being the major cancers in Japan; or from a report on guidelines for cancer survivors by the American Cancer Society aimed at improving prognosis^5^. Total caloric intake was analyzed as the amount of food consumed, and sodium intake as the result of intake of various salted foods, or salty cooking behavior. In addition, green tea and coffee were added based on a cancer risk assessment for Japanese^20^, and rice and miso soup were included as foundational items of the traditional Japanese diet^21^. Participants within the study who developed cancer were analyzed before and after diagnosis, while those who did not develop cancer served as controls.

## Materials and methods

### Study population

The Japan Public Health Center-based Prospective Study (JPHC study) was started in 1990 (cohort I) and 1994 (cohort II) in 11 public health center (PHC) areas. Participants (n = 140,420) were aged 40–69 years at the time of the initial self-administered surveys. Enrolled individuals were informed of the objectives and contents of the study. Informed consent was obtained from each participant implicitly when they completed the questionnaires. After the initial survey, 5-year and 10-year follow-up self-administered surveys were conducted in 1995–1998 and 2000–2003, respectively, to update the information on diet, lifestyle habits, and health condition of these individuals. Details of the JPHC study have been described previously^22^. Because the questionnaires used in the 5-year and 10-year follow-up surveys requested more comprehensive information on dietary intake than the initial survey, this study used data from the 5- year and 10-year surveys for the baseline and follow-up measurements, respectively. Hereafter in this manuscript, the 5-year survey will be referred to as the baseline survey and the 10-year survey as the follow-up survey. Exclusion criteria were non-Japanese nationality, incorrect birth date, multiple registrations, or leaving the area before the start of the study (n = 275); lack of complete data on cancer incidence (n = 7078; all in Katsushika in Tokyo prefecture);death, moving out, or loss or refusal to follow-up before the baseline surveys (n = 12,062); and cancer diagnosis before the baseline survey (n = 2389). We further excluded persons with a self-reported history of cancer at the baseline survey (n = 637) or who had died, moved out, or were lost to or refused follow-up before the follow-up surveys (n = 3719). The remaining 81,896 participants yielded a response rate of 81.4% for the baseline survey and 88.8% for the follow-up survey. Of 81,896 study participants, 1,191 with incomplete responses on diet and 3,138 with missing information on ethanol intake were excluded. Participants (n = 1,516) with missing or implausible values for body mass index (BMI) as a potential confounder (< 100 cm or > 199 cm for height or < 20 kg for body weight) were excluded from the analysis. We further excluded 2,922 participants who reported implausible energy intake at baseline and follow-up surveys (lower and upper 1st percentile, 884 and 5,024 kcal/day for men and 741 and 4,440 kcal/day for women, respectively, for the baseline and 729 and 5,189 kcal/day for men and 596 and 4,668 kcal/day for women, respectively, for the follow-up survey). In this survey, percentile values were employed so that the same number of people were excluded by the upper and lower percentiles to avoid biasing exclusion toward those with low intakes only. In addition, to ensure the scale of the survey despite exclusions based on two surveys, we defined possible serious errors as the lower or upper 1 percentile^23^ (looser inclusion criteria). Finally, the study included 73,192 participants (33,643 men and 39,549 women). The study was approved by the Institutional Review Boards of the National Cancer Center in Tokyo, Japan (approval number: 2001-021), and all study procedures were performed in accordance with the relevant guidelines and regulations in Japan.

### Food frequency questionnaires

We examined the changes in calories, 2 nutrients, and in 16 foods and food groups before and after the cancer diagnosis, and compared results with the cancer-free control participants. These dietary intakes were estimated from validated food frequency questionnaires (FFQs) used for the baseline and follow-up surveys, whose validity for the estimation of nutrient and food groups has been documented^24–28^. The FFQs asked about usual consumption using 138 food and beverage items during the previous year. The questionnaire contained nine frequency categories for food items ranging from “almost never” to “seven or more times per day.” Nine frequency choices for beverages ranged from “almost never” to “10 or more glasses per day.” Standard portion sizes were specified for each food item in three amount choices: small (50% smaller than standard), medium (standard) and large (50% larger). The amount of foods and beverages consumed (g/day) was calculated from the responses. Energy and nutrient intake were calculated using the Standard Tables of Food Composition in Japan 2015 (7th Revised Edition)^29^.

Spearman’s rank correlation coefficients for food groups between energy-adjusted nutrient intake based on the FFQ and those based on 28-day (or 14-day for one public health center area in Okinawa) dietary records among subsamples of the cohorts^24–28^. The coefficients for food groups ranged from 0.22 (vegetables) to 0.75 (coffee) for men and from 0.22 (grains) to 0.80 (coffee) for women in cohort I and cohort II.

### Information on survival status and cancer diagnosis

Information on survival status, including survival between the baseline and follow-up survey, was collected annually from the residential registers from each municipality in the study area. Death certificates for persons in the residential registry are forwarded to the Japanese Ministry of Health Labor and Welfare, and are coded for inclusion in the national Vital Statistics. Residency registration and death registration are required by the Basic Residential Register Law and Family Registry Law, respectively, and are thought to be complete. The occurrence of cancer was confirmed from the following two data sources: active patient notification from major local hospitals in the study area, and data linkage with population-based cancer registries with the permission of the local governments responsible for the cancer registries. Cases of cancer were coded according to the International Classification of Diseases for Oncology, Third Edition^30^. In our cancer registry system of present study, the proportion of cases for which information was available from death certificates only was 0.8%. Participants with any cancer diagnosed between study periods were defined as survivors in this study. Site-specific cancers (C18, C19, and, C20 as colorectal, C16 as gastric, and C50 as breast cancers) that are major cancers in Japan were also analyzed separately. If a participant was diagnosed with more than one cancer, the cancer that had the earlier diagnosis was used for the analysis.

### Statistical analysis

Individual changes in dietary intake were calculated by subtracting intake values at baseline from those at follow-up based on the respective FFQ. The Wilcoxon signed-rank test was used to determine the significance of any change of intake between baseline and follow-up survey. Changes among cancer survivors were compared with those among cancer-free the controls. The Mann–Whitney U-test was used to compare controls and survivors for all cancers, and the Kruskal–Wallis test was used for controls and survivors for specific cancer types. Because this comparison of controls and survivors for specific cancer types was conducted by univariate analysis, multiple comparisons were not performed. Statistical significance was considered at the *P *< 0.01 level in this analysis because of the large number of participants. For these univariate analyses, Bonferroni correction (with statistical significance considered at the *P *< 0.0005 level for both genders) was further made because of the number of exposures. Only those items for which significant differences were found in these univariate analyses were considered in further multivariate analysis. Relative changes in energy-adjusted intake (/1000 kcal by the density method), expressed as the ratio of the follow-up intake value to the baseline intake value as the dependent variable, were calculated using linear regression analysis with the existence of a cancer diagnosis as the independent variable. Those with zero intake in the baseline survey only were excluded, because it was impossible to calculate from each item as follows: for men, ethanol (n = 1473, 3.0% of survivors, 4.4% of controls); rice (n = 261, 0.3% of survivors, 0.8% of controls); and milk and dairy products (n = 1117, 4.3% of survivors, 3.3% of controls) were excluded. For women, rice (n = 344, 0.6% of survivors, 0.9% of controls) and vegetables (n = 102, 0.2% of survivors, 0.3% of controls) were excluded. These relative changes were calculated by the following formula:

(energy-adjusted intakes by follow-up survey − those by baseline survey)/energy-adjusted intakes by baseline survey.

Multiple linear regression analysis was carried out to adjust for the following potential confounders: age (continuous), PHC area (10 areas), BMI (< 18.5, 18.5–24.9, 25–29.9, or ≥ 30), living alone (yes/no), physical activity in metabolic equivalent task-hours/day (< 30, 30–34.9, 35–39.9, ≥ 40, or missing), smoking status (never, past, current < 20, or ≥ 20 cigarettes/d), and quintiles of energy intake at baseline survey. *P* values were 2-sided, and statistical significance was determined at *P *< 0.05. All analysis was conducted using commercial software (SAS version 9.4; SAS Institute Inc., Cary NC, USA).

### Ethics statement

Enrolled individuals were informed of the objectives and contents including follow-up, of the study. Informed consent was obtained from each participant implicitly when they completed the questionnaires of the JPHC Study. The study was approved by the Institutional Review Boards of the National Cancer Center in Tokyo, Japan (approval number: 2001-021), and all procedures in this study were performed in accordance with the relevant ethical guidelines regulations in Japan. Detailed information on the study was mailed to each participant and is published on the study website (http://epi.ncc.go.jp/jphc).

## Results

We confirmed 1,532 cases of newly diagnosed cancer (886 cases among men and 646 cases among women) among the 73,192 participants by December 31, 2002. By site, these were 226 cases of colorectal, 244 cases of gastric, and 416 cases of other cancers among men, and 134 cases of colorectal, 91 cases of gastric, 158 cases of breast, and 263 cases of other cancers among women. Mean duration between diagnosis and follow-up dietary measurement was 2.1 years for the survivors.

### Characteristics of participants

The baseline characteristics of participants are shown in Table 1. With the exception of breast cancer patients, survivors were older than the cancer-free control participants among both men and women. The distribution by BMI category was similar in controls and survivors other than for gastric cancer in men (relatively low proportion of overweight) and for breast cancer (relatively high proportion of obesity) in women. There were more former smokers among survivors than controls, while nonsmokers were less common among survivors than among controls. The proportion of participants living alone was similar in both populations, although with a relatively lower proportion of men in gastric cancer survivors.

**Table 1:** Characteristics of participants according to cancer diagnosis and specific cancer site: the Japan Public Health Center-based Prospective Study, 1995 and 1998 (n = 73,192).

	Men (n = 33,643)					Women (n = 39,549)					
	Cancer-free controls	All cancers	Major site-specific cancers (n = 886)			Cancer-free controls	Total cancers	Major site-specific cancers (n = 646)			
			Colorectal cancer	Gastric cancer	Other cancers			Colorectal cancer	Gastric cancer	Breast cancer	Other cancers
Number of participants	32,757	886	226	244	416	38,903	646	134	91	158	263
Age (mean ± SD)	56.4 ± 7.6	61.5 ± 7.1	60.2 ± 7.0	61.8 ± 6.5	62.1 ± 7.3	56.8 ± 7.6	58.0 ± 7.9	59.4 ± 7.4	60.0 ± 7.4	55.8 ± 7.1	58 ± 8.4
Body mass index (kg/m^**2**^**, %)**											
< 18.5	2.5	3.4	3.1	2.5	4.1	3.5	3.1	3.0	4.4	3.2	2.7
≥ 18.5, < 25	67.7	69.8	69.5	75.8	66.3	67.1	64.9	62.7	69.2	63.9	65.0
≥ 25, < 30	27.5	25.4	27.0	20.1	27.6	26.1	28.6	32.1	24.2	28.5	28.5
≥ 30	2.2	1.5	0.4	1.6	1.9	3.3	3.4	2.2	2.2	4.4	3.8
Physical activity (MET–h/d, %)											
< 30	38.0	38.8	38.5	34.8	41.3	33.7	36.4	34.3	39.6	25.9	42.6
≥ 30, < 35	28.8	30.7	32.3	32.4	28.8	44.4	43.0	44.8	38.5	56.3	35.7
≥ 35, < 40	7.8	7.4	9.3	6.1	7.2	6.2	5.7	4.5	6.6	5.1	6.5
≥ 40	22.6	20.4	17.7	22.1	20.9	13.1	11.8	11.2	14.3	11.4	11.4
Missing	2.8	2.6	2.2	4.5	1.7	2.6	3.1	5.2	1.1	1.3	3.8
Smoking status (%)											
Past smoker	18.0	24.3	25.7	19.3	26.4	0.9	0.9	1.5	0.0	1.3	0.8
Never smoked	35.0	28.0	25.7	29.5	28.4	91.0	89.0	88.1	87.9	89.9	89.4
Current smoker	46.0	46.6	48.2	49.6	44.0	4.9	6.8	5.2	9.9	7.6	6.1
Missing	1.0	1.1	0.4	1.6	1.2	3.2	3.3	5.2	2.2	1.3	3.8
Living alone (%)	2.5	2.7	2.7	0.8	3.8	5.7	5.6	5.2	11	3.2	5.3
Public health center area (%)											
Ninohe	10.7	6.0	7.1	5.3	5.8	11.5	11.0	9.0	9.9	14.6	10.3
Yokote	13.8	14.4	19.9	17.2	9.9	14.5	17.2	24.6	19.8	15.8	13.3
Saku	12.4	13.4	13.7	15.6	12.0	11.4	14.4	12.7	11.0	12.7	17.5
Okinawa	8.0	4.4	6.6	1.6	4.8	7.6	7.7	5.2	4.4	13.3	6.8
Mito	20.5	22.6	16.4	29.5	21.9	18.8	16.4	14.2	15.4	17.1	17.5
Nagaoka	3.1	5.1	7.1	6.1	3.4	3.0	4.3	5.2	6.6	1.3	4.9
Kochi	7.7	5.5	5.8	6.6	4.8	7.8	6.5	5.2	3.3	8.2	7.2
Nagasaki	8.2	14.7	11.1	11.1	18.8	9.2	10.4	11.2	16.5	5.7	10.6
Miyako	9.9	8.4	6.2	2.9	12.7	9.9	7.4	7.5	7.7	5.7	8.4
Suita	5.7	5.5	6.2	4.1	6.0	6.4	4.6	5.2	5.5	5.7	3.4

*MET– h* Metabolic equivalent task–hours.

### Comparison of changes in crude intakes

Crude intakes of energy, nutrients, and food groups estimated by FFQs and those changes over five years are shown in Table 2 for men and Table 3 for women, respectively. Changes in nutrient intakes as a result of changes in food habits are shown in Supplemental tables S1 and S2. At baseline, intake levels in survivors were similar to those in the cancer-free controls among both men and women. Most nutrients and food groups examined showed significant changes between baseline and follow-up surveys for both cancer-free controls and survivors of all cancers among both sexes. Most nutrients or food groups showed no statistically significant difference in the change in intake between cancer-free controls and survivors for either gender. In men, total energy intake decreased in both survivors and controls over five years, with a significantly greater decrement in survivors (median change: − 168 kcal**/**d [Interquartile range {IQR}: − 640, 278]) than controls (− 33 kcal**/**d [IQR: − 453, 380], *P* < 0.001). Reductions in total energy intake in male gastric cancer survivors were the largest among all survivors of site-specific cancers (gastric cancer: − 255 kcal**/**d [IQR: − 810, 168], colorectal cancer: − 159 kcal/d [− 581, 268]; other cancers: − 115 kcal/d [− 571, 335], *P* < 0.001). Also, ethanol intake decreased significantly more in male survivors than in cancer-free controls, with corresponding median [IQR] and *P* values of − 1.5 g/d [− 23.0, 0], 0 g/d [− 9.0, 5.0], and *P* < 0.001, respectively, for ethanol. Rice intake increased both in survivors of all cancers (34.7 g/d [− 88.3, 104.3]) and controls (64.5 g/d [− 55.0, 134.6]), with significantly smaller changes in survivors than controls (*P* < 0.001). The results for grains were similar to those for rice, which accounts for the major part of grain intake. Milk and dairy product or coffee intake did not materially change between the baseline and follow-up surveys in survivors of all cancers and controls, although these did show statistical significance (*P *< 0.01 for both). In further Bonferroni correction, the statistical significance of milk and dairy products or coffee was lost, whereas the results for total energy, ethanol and rice were not changed. Additionally, the reduction in energy intake among male survivors was larger for short-period (< 1 year from the diagnosis to the follow-up dietary measurement) survivors than for longer-period (> 4 years) survivors: corresponding median changes [IQR] for short- and longer-period survivors were − 159 kcal/d [− 596, 284] and − 98 kcal/d [− 558, 343], respectively) Also, the reduction in ethanol intake among male survivors was slightly greater for the short-period survivors (− 1.7 g/d [− 27.1, 0]) than for the longer-period survivors (0 g/d, [− 23.0, 0.4]).

**Table 2:** Changes in crude intakes of energy, nutrients and food groups from baseline for male cancer survivors compared with male controls (n = 33,643).

	Cancer-free controls (n = 32757)			All cancers (n = 886)				*P*^a^	Colorectal cancer (n = 226)			Gastric cancer (n = 244)			Other cancers (n = 416)			*P*^b^
	Baseline median	Change from baseline		Baseline median	Change from baseline				Baseline median	Change from baseline		Baseline median	Change from baseline		Baseline median	Change from baseline		
		median	IQR		median		IQR			median	IQR		median	IQR		median	IQR	
Energy and nutrients																		
Total energy (kcal/d )	2110	− 33	(− 453–380)*	2127	− 168		(− 640–278)*	** <0 .001**	2257	− 159	(− 581–268)*	2151	− 255	(− 810–168)*	2063	− 115	(− 571–335)*	** < .001**
Sodium (mg/d )	4603	− 248	(− 1555–1003)*	4627	− 345		(− 1723–1110)*	0.41	4673	− 368	(− 1599–906)	4759	− 369	(− 1811–857)*	4465	− 335	(− 1681–1224)	0.61
Ethanol (g/d)	23.0	0.0	(− 9.0–5.0)*	23.0	− 1.5		(− 23.0–0.0)*	** <0 .001**	36.0	0.0	(− 23.0–0.0)*	23.0	− 7.9	(− 26.4–0.0)*	14.8	0.0	(− 18.5–0.0)*	** < .001**
Food groups (g/d)																		
Grains	570.9	30.6	(− 98.9–139.1)*	562.6	− 11.3		(− 148.9–111.5)	** <0 .001**	572.9	7.4	(− 132.9–109.5)	580.4	-56.3	(− 200.2–91.9)*	546.8	0.9	(− 136.1–117.8)	** <0 .001**
Rice	423.3	64.5	(− 55.0–134.6)*	423.3	34.7		(− 88.3–104.3)*	** < 0.001**	423.3	54.6	(− 79.8–135.0)*	425.0	− 7.0	(− 157.3–91.3)	423.3	37.5	(− 80.0–101.1)*	** < 0.001**
Soy products	30.7	0.0	(− 17.0–19.1)*	31.4	0.0		(− 17.1–21.3)	0.58	33.9	− 0.3	(− 15.1–20.2)	29.7	− 1.6	(− 19.2–18.7)	31.1	0.9	(− 16.9–23.5)	0.36
Miso soup	235.7	0.0	(− 80.4–58.9)*	235.7	0.0		(− 88.4–75.0)	0.95	225.0	0.0	(− 75.0–80.4)	300.0	0.0	(− 112.5–75.0)	225.0	0.0	(− 85.7–62.9)	0.54
Vegetables	170.8	7.9	(− 65.8–87.7)*	180.6	12.0		(− 69.2–99.9)*	0.42	195.3	14.7	(− 77.5–104.8)	193.6	9.3	(− 81.4–103.4)	173.7	13.4	(− 62.8–93.4)	0.76
Pickled vegetables	20.5	0.0	(− 11.8–12.8)	24.5	− 0.9		(− 16.2–13.3)	0.13	27.2	0.0	(− 15.0–13.2)	27.6	− 2.0	(− 19.9–14.5)	21.1	− 0.7	(− 14.0–12.4)	0.33
Fruit	141.4	− 13.6	(− 92.9–58.4)*	151.1	− 3.1		(− 90.1–85.0)	0.01	147.0	− 3.4	(− 83.2–71.0)	155.0	7.3	(− 84.6–106.5)	151.2	− 7.7	(− 97.0–72.4)	0.04
Fish and shellfish	79.3	− 15.3	(− 49.9–16.6)*	79.8	− 16.0		(− 55.0–17.6)*	0.54	84.0	− 14.7	(− 51.8–16.4)*	80.3	− 20.8	(− 61.5–18.9)*	76.8	− 15.6	(− 53.6–17.6)*	0.66
Salty fish	14.0	− 1.5	(− 12.0–4.2)*	15.4	− 2.0		(− 13.8–5.2)*	0.95	15.5	− 3.0	(− 14.3–4.0)*	18.9	− 2.2	(− 16.4–6.5)*	13.0	− 1.0	(− 11.9–5.2)*	0.92
Meat	52.7	− 4.3	(− 31.1–25.2)*	47.1	− 5.3		(− 29.1–19.3)*	0.32	53.2	− 8.3	(− 37.6–14.0)*	45.8	− 6.6	(− 30.2–17.3)*	45.1	− 2.2	(− 25.9–28.2)	0.02
Unprocessed red meat	36.7	− 7.3	(− 28.1–11.7)*	34.3	− 7.3		(− 28.1–8.9)*	0.37	41.0	− 13.7	(− 33.5–4.9)*	31.9	− 7.6	(− 27.5–6.0)*	33.3	− 5.1	(− 25.5–12.3)*	0.02
Processed meat	3.5	− 0.7	(− 4.2–1.7)*	3.0	− 0.5		(− 3.3–1.3)*	0.66	3.3	− 0.3	(− 3.3–1.5)	3.0	− 0.8	(− 3.3–1.2)*	3.0	− 0.7	(− 3.2–1.5)*	0.90
Milk and dairy products	112.5	− 1.3	(− 65.6–52.8)*	119.9	0.0		(− 74.3–83.5)	** < 0.01**	108.6	5.7	(− 52.7–86.5)	128.5	− 0.8	(− 97.5–76.2)	118.6	1.1	(− 77.8–85.1)	** < 0**.**01**
Green tea	120.0	0.0	(− 120.0–120.0)*	300.0	0.0		(− 240.0–180.0)	0.40	300.0	0.0	(− 300.0–180.0)	300.0	0.0	(− 300.0–180.0)	120.0	0.0	(− 180.0–205.7)	0.15
Coffee	113.6	0.0	(− 79.3–34.3)*	79.3	0.0		(− 79.3–0.0)*	** <0 .01**	60.0	0.0	(− 60.0–15.0)*	79.3	0.0	(− 89.3–0.0)*	79.3	0.0	(− 79.3–0.0)*	0.04
Soft drinks	42.9	0.0	(− 53.6–0.0)*	42.9	0.0		(− 53.6–0.0)*	0.96	42.9	0.0	(− 57.1–0.0)*	42.9	0.0	(− 42.9–0.0)*	0.0	0.0	(− 53.6–0.0)*	0.09

*IQR* Interquartile range

*Statistical significance was set at *P *< .01 and the Wilcoxon signed − rank test was used to determine the significance of differences in intake between the baseline and follow-up surveys.

^a^The Mann–Whitney *U* − test was used for the difference in change between cancer-free male controls and survivors of all cancers, ^b^The Kruskal − Wallis test was used for the comparison of the change among cancer-free male controls and survivors of specific cancer types. Statistical significance was set at *P *< .01 for both test.

Significant values are in [bold].

**Table 3:** Changes in crude intakes of energy, nutrients and food groups from baseline for female cancer survivors compared with female controls (n = 39,549).

	Cancer-free controls (n = 38,903)				All cancers (n = 646)				Colorectal cancer (n = 134)			Gastric cancer (n = 91)			Breast cancer (n = 158)			Other cancers (n = 263)				*P*^b^
	Baseline median	Change from baseline			Baseline median	Change from baseline		*P*^a^	Baseline median	Change from baseline		Baseline median	Change from baseline		Baseline median	Change from baseline		Baseline median	Change from baseline			
		median	IQR			median	IQR			median	IQR		median	IQR		median	IQR		median	IQR		
Energy and nutrients																						
Energy (kcal/d)	1800	− 72	(− 422–278)*		1788	− 90	(− 421–255)*	0.69	1779	− 25	(− 322–365)	1851	− 283	(− 542–161)*	1820	3	(− 317–268)	1757	− 109	(− 444–226)*		0.04
Sodium (mg/d)	4496	− 257	(− 1464–904)*		4559	− 173	(− 1408–1060)	0.18	4658	− 14	(− 1212–1442)	4741	− 431	(− 1953–905)	4226	− 105	(− 1123–1201)	4565	− 296	(− 1448–938)		0.07
Ethanol (g/d)	0.0	0.0	(0.0–0.0)*		0.0	0.0	(0.0–0.0)	0.11	0.0	0.0	(0.0–0.0)	0.0	0.0	(0.0–0.0)	0.0	0.0	(0.0–0.0)	0.0	0.0	(0.0–0.0)		0.47
Food groups (g/day)																						
Grains	486.1	− 18.0		(− 111.1–60.3)*	486.7	− 34.2	(− 131.4–39.8)*	** < 0.01**	486.5	− 15.9	(− 106.1–51.7)	489.4	− 94.7	(− 220.7–59.2)*	492.6	− 25.8	(− 131.0–32.5)*	480.9	− 26.9	(− 131.1–36)*		** < 0.01**
Rice	367.5	0.0		(− 53.9–63.2)*	420.0	− 3.3	(− 90.0–26.8)*	** < 0.01**	420.0	− 1.7	(− 60.0–25.0)	420.0	− 19.9	(− 140.0–31.7)*	331.4	− 3.3	(− 60.7–39.6)	420.0	− 2.3	(− 64.0–18.0)		** < 0.01**
Soy products	35.1	0.2		(− 16.0–22.4)*	36.3	0.0	(− 20.1–23.8)	0.50	35.4	4.8	(− 17.9–35.0)	43.3	− 5.5	(− 30.0–20.7)	36.0	5.6	(− 13.8–32.3)*	35.7	− 1.6	(− 20.6–17.1)		0.01
Miso soup	176.8	0.0		(− 75.0–42.9)*	225.0	0.0	(− 75.0–42.9)	0.79	225.0	0.0	(− 75.0–56.3)	225.0	0.0	(− 85.7–75.0)	150.0	0.0	(− 75.0–50.9)	225.0	0.0	(− 64.3–37.5)		0.78
Vegetables	205.5	15.5		(− 63.6–102.9)*	201.4	27.3	(− 45.9–124.7)*	** <0 .01**	196.6	29.3	(− 43.6–127.5)	248.4	21.6	(− 73.5–98.1)	192.6	30.7	(− 37.5–144.5)*	202.2	22.1	(− 49.1–121.9)*		0.02
Pickled vegetables	23.4	0.0		(− 13.5–14.8)*	25.6	0.0	(− 15.4–17.1)	0.72	24.9	4.4	(− 19.0–25.5)	35.7	− 4.4	(− 29.6–19.8)	23.2	0.8	(− 12.2–15.2)	24.8	− 0.2	(− 13.0–15.6)		0.58
Fruit	207.1	− 15.6		(− 110.8–79.4)*	210.1	− 15.3	(− 118.4–90.0)	0.70	204.5	15.2	(− 110.9–114.1)	250.8	− 26.1	(− 131.5–120.4)	220.3	− 24.2	(− 122.8–91.0)	196.9	− 19.4	(− 124.2–79.7)		0.65
Fish and shellfish	77.6	− 13.5		(− 44.8–16.2)*	76.3	− 12.1	(− 45.4–15.3)*	0.77	78.5	− 12.5	(− 39.8–16.2)	81.7	− 24.4	(− 63.6–9.8)*	72.0	− 9.5	(− 40.2–16.8)*	73.7	− 7.8	(− 45.9–16.4)*		0.33
Salty fish	15.2	− 1.3		(− 11.1–4.7)*	15.0	− 1.7	(− 11.2–2.5)*	0.16	19.5	− 1.0	(− 12.5–4.3)	13.5	− 4.7	(− 19.5–0.0)*	14.1	− 1.4	(− 9.0–2.5)*	13.0	− 1.5	(− 11.0–2.7)*		0.11
Meat	46.7	− 2.6		(− 26.0–24.2)*	46.7	-3.0	(− 26.8–22.2)	0.61	45.1	0.5	(− 25.4–24.7)	47.1	− 8.7	(− 34.8–12.7)	48.4	− 5.4	(− 25.4–31.4)	44.6	− 3.3	(− 26.0–20.8)		0.43
Unprocessed red meat	32.1	− 6.0		(− 24.4–9.9)*	31.7	− 7.0	(− 25.8–8.2)*	0.41	30.2	− 4.8	(− 19.4–10.9)	32.4	− 9.3	(− 29.7–3.9)*	32.9	− 9.0	(− 26.6–11.3)*	31.4	− 6.7	(− 24.9–6.3)*		0.60
Processed meat	3.8	− 0.7		(− 3.8–1.6)*	3.5	− 0.6	(− 3.5–1.5)*	0.75	4.3	− 1.0	(− 4.6–1.9)	3.2	− 0.5	(− 4.4–1.2)*	3.8	− 0.9	(− 3.5–1.0)*	3.3	− 0.2	(− 3.3–2.2)		0.60
Milk and dairy products	176.5	− 1.5		(− 81.4–68.1)*	193.2	0.0	(− 85.2–86.7)	0.25	200.0	30.4	(− 57.1–120.7)	200.0	− 14.1	(− 121.8–96.8)	192.7	5.3	(− 60.3–88.5)	180.4	− 2.8	(− 92.6–63.2)		0.08
Green tea	300.0	0.0		(− 120.0–180.0)*	300.0	0.0	(− 180.0–205.7)	0.88	300.0	0.0	(0.0–300.0)	300.0	0.0	(− 300.0–274.3)	300.0	0.0	(− 180.0–274.3)	300.0	0.0	(− 180.0–120.0)		0.12
Coffee	79.3	0.0		(− 53.6–25.7)*	60.0	0.0	(− 53.6–0.0)*	0.11	25.7	0.0	(− 53.6–0.0)	25.7	0.0	(− 60.0–0.0)	79.3	0.0	(− 53.6–25.7)	25.7	0.0	(− 53.6–0.0)		0.52
Soft drinks	0.0	0.0		(− 42.9–0.0)*	0.0	0.0	(− 42.9–0.0)*	0.32	0.0	0.0	(− 42.9–0.0)	0.0	0.0	(− 42.9–0.0)	0.0	0.0	(− 42.9–0.0)*	0.0	0.0	(− 42.9–0.0)*		0.34

*IQR* Interquartile range

*Statistical significance was set at *P *< .01 and the Wilcoxon signed − rank test was used to determine the significance of differences in intake between the baseline and follow-up surveys.

^a^The Mann–Whitney *U* − test was used for the difference in change between cancer-free male controls and survivors of all cancers, ^b^The Kruskal − Wallis test was used for the comparison of the change among cancer-free female controls and survivors of specific cancer types. Statistical significance was set at *P* < .01 for both test.

Significant values are in [bold]

In women, total energy intake was decreased in both survivors (− 90 kcal**/**d [− 421, 255]) and controls (− 72 kcal**/**d [− 422, 278]), but not to a significant extent (*P* = 0.69). Additionally, after adjustment for age and other factors, the relative changes in energy intake among women also showed no difference between cancer survivors and cancer-free participants (1% [95% confidence intervals (CI): − 2 to 4]). Rice intake was decreased in female survivors (− 3.3 g/d [− 90.0, 26.8] for all cancers), especially survivors of gastric cancers (gastric cancer: − 19.9 g/d [− 140.0, 31.7], colorectal cancer: − 1.7 g/d [− 60.0, 25.0], breast cancer: − 3.3 g/d [− 60.7, 39.6], and other cancers: − 2.3 g/d [− 64.0, 18.0]) in contrast to cancer-free controls (0 g/d [− 53.9, 63.2], *P* < 0.01). The results for grains were similar to those for rice. Intake of vegetables, but not of fruit, was increased more in female survivors (27.3 g/d [− 45.9, 124.7]) vs. controls (15.5 g/d [− 63.6, 102.9]) (*P* < 0.01), especially in survivors of breast cancer (breast cancer: 30.7 g/d [− 37.5, 144.5], colorectal cancer: 29.3 g/d [− 43.6, 127.5], gastric cancer: 21.6 g/d [− 73.5, 98.1], and other cancers: 22.1 g/d [− 49.1, 121.9], *P* = 0.02). On further Bonferroni correction, the statistical significance for grains and vegetables was lost, whereas the results for rice among survivors of all cancers and controls only were not changed. There was no difference in the change in red meat intake between survivors and controls of either gender (male survivors of all cancers and controls were − 7.3 g/d [− 28.1, 8.9] and − 7.3 g/d [− 28.1, 11.7], respectively, *P* = 0.37, female survivors and controls were: − 7.0 g/d [− 25.8, 8.2] and − 6.0 g/d [− 24.4, 9.9], respectively, *P* = 0.41).

### Comparison in relative changes in energy-adjusted intakes

Relative changes in nutrient and food group intakes for cancer survivors compared with those for cancer-free controls are shown in Table 4 for men and women. Energy-adjusted intake was used because the energy intake was decreased for both cancer survivors and controls in both genders. We analyzed the nutrients and the food groups that showed statistically significant differences in the prior comparison by crude intake: ethanol, grains, rice, milk and dairy products in men; and grains, rice and vegetable in women. Compared to controls, a significant decrement (β =  − 0.36, [95% CI: − 0.53 to − 0.20]), especially in gastric cancer survivors (β =  − 0.45, [95% CI: − 0.76 to − 0.14]), was observed in ethanol intake among men. Among women, changes in intake between baseline and follow-up did not differ between cancer survivors and controls in the fully-adjusted models.

**Table 4:** Relative changes^a^ in energy-adjusted nutrient and food group intakes in cancer survivors compared with controls for male (n = 33,643) and female (n = 39,549).

	Model 0			Model 1		Changes in ratio to the baseline intake		Absolute changes in intake	
	N	β	CI	β	CI	Mean	SD	Mean	SD
***Male***									
**Ethanol**^b^	32,170								
Cancer–free controls		REF		REF		0.25	2.46	− 0.9	11.7
Cancer		** − 0.44**	**(− 0.61** to ** − 0.28)**	** − 0.36**	**(− 0.53** to ** − 0.20)**	− 0.19	1.06	− 5.3	13.5
Colorectal cancer		** − 0.41**	**(− 0.74** to ** − 0.08)**	** − 0.36**	**(− 0.68** to ** − 0.03)**	− 0.15	0.66	− 5.2	14.8
Gastric cancer		** − 0.55**	**(− 0.86** to ** − 0.24)**	** − 0.45**	**(− 0.76** to ** − 0.14)**	− 0.30	1.05	− 6.1	12.3
Other cancer		** − 0.39**	**(− 0.63** to ** − 0.16)**	** − 0.32**	**(− 0.56** to ** − 0.08)**	− 0.14	1.22	− 4.8	13.4
**Grains**^b^	33,643								
Cancer–free controls		REF		REF		0.12	0.46	16.9	92.3
Cancer		0.01	(− 0.02–0.04)	0.00	(− 0.03–0.03)	0.13	0.50	16.2	96.9
Colorectal cancer		0.02	(− 0.04–0.09)	0.02	(− 0.04–0.08)	0.15	0.43	22.6	92.4
Gastric cancer		− 0.01	(− 0.07–0.05)	− 0.01	(− 0.07–0.05)	0.12	0.53	9.7	98.2
Other cancer		0.01	(− 0.03–0.06)	0.00	(− 0.04–0.05)	0.14	0.52	16.5	98.6
**Rice**^b^	33,382								
Cancer–free controls		REF		REF		2.38	19.56	27.6	94.0
Cancer		0.43	(− 0.88–1.73)	0.22	(− 1.10–1.53)	2.81	19.00	27.8	99.9
Colorectal cancer		− 0.11	(− 2.67–2.46)	− 0.28	(− 2.84–2.28)	2.27	13.16	35.8	89.4
Gastric cancer		1.99	(− 0.47–4.46)	1.86	(− 0.60–4.33)	4.37	27.18	21.0	107.2
Other cancer		− 0.20	(− 2.09–1.69)	− 0.48	(− 2.37–1.41)	2.18	15.54	27.4	100.8
**Milk and dairy products**^b^	32,526								
Cancer–free controls		REF		REF		2.26	25.04	− 3.9	99.9
Cancer		1.08	(− 0.62–2.78)	1.08	(− 0.63–2.79)	3.34	19.64	9.4	119.0
Colorectal cancer		0.83	(− 2.52–4.18)	0.73	(− 2.62–4.09)	3.09	15.50	13.4	112.8
Gastric cancer		0.65	(− 2.57–3.86)	0.66	(− 2.56–3.89)	2.90	13.25	3.7	137.7
Other cancer		1.46	(− 0.99–3.91)	1.50	(− 0.96–3.96)	3.72	24.20	10.5	110.4
***Female***									
**Grains**^b^	39,549								
Cancer–free controls		REF		REF		0.06	0.42	0.3	88.2
Cancer		** − 0.04**	**(− 0.07** to ** − 0.01)**	** − 0.04**	**(− 0.07** to ** − 0.01)**	0.01	0.40	− 10.7	90.5
Colorectal cancer		** − **0.05	(− 0.12 − 0.03)	− 0.04	(− 0.11–0.03)	0.01	0.34	-9.9	90.4
Gastric cancer		** − **0.03	(− 0.12–0.05)	− 0.04	(− 0.13–0.04)	0.02	0.63	− 18.8	121.7
Breast cancer		** − **0.07	(− 0.13–0.00)	− 0.06	(− 0.12–0.01)	− 0.01	0.35	− 14.0	74.7
Other cancer		** − **0.03	(− 0.08–0.02)	− 0.03	(− 0.08–0.02)	0.03	0.36	− 6.4	86.8
**Rice**^b^	39,205								
Cancer–free controls		REF		REF		2.56	17.76	11.9	90.7
Cancer		− 1.19	(− 2.57–0.19)	− 1.13	(− 2.50–0.24)	1.36	10.16	− 1.6	88.5
Colorectal cancer		− 1.84	(− 4.85–1.17)	− 1.67	(− 4.65–1.32)	0.72	7.67	− 9.3	94.2
Gastric cancer		− 1.31	(− 4.97–2.34)	− 1.23	(− 4.86–2.39)	1.24	11.06	− 12.0	96.8
Breast cancer		− 1.05	(− 3.82–1.72)	− 1.10	(− 3.85–1.65)	1.50	9.97	2.6	82.6
Other cancer		− 0.91	(− 3.05–1.24)	− 0.84	(− 2.97–1.29)	1.65	11.06	3.2	85.8
**Vegetables**^b^	38,802								
Cancer–free controls		REF		REF		0.38	1.76	17.3	89.3
Cancer		0.11	(− 0.02–0.25)	0.11	(− 0.03–0.24)	0.49	1.15	33.1	102.3
Colorectal cancer		0.10	(− 0.20–0.39)	0.10	(− 0.20–0.39)	0.48	1.01	29.4	88.2
Gastric cancer		0.10	(− 0.26–0.46)	0.11	(− 0.25–0.47)	0.48	1.06	32.8	125.5
Breast cancer		0.13	(− 0.15–0.40)	0.12	(− 0.15–0.40)	0.51	1.10	40.9	106.9
Other cancer		0.12	(− 0.09–0.33)	0.11	(− 0.11–0.32)	0.50	1.28	30.4	97.6

β: Difference in the intake changes compared with cancer–free controls, CI: 95% confidence intervals.

^a^ Changes are expressed as the ratio to the baseline intake according to following formula: (follow–up survey intake − baseline survey intake)/baseline survey intake; intakes were energy − adjusted by the density method.

^b^ The unit of intake is g/day.

Model 0: Univariate model.

Model 1: Model was adjusted for age (continuous), public health center area, body mass index in kg/m^2^ (< 18.5, 18.5 − 24.9, 25 − 29.9, ≥ 30), living alone (yes/no), physical activity in metabolic equivalent task − hours/day (< 30, 30–34.9, 35–39.9, and ≥ 40), smoking status (never, past, current < 20, and current ≥ 20 cigarettes/d), and quintile of energy intake.

Significant values are in [bold].

## Discussion

This study investigated dietary changes in cancer survivors compared to cancer-free controls by gender based on a large-scale prospective cohort study in Japan. Although there was a marked decrease in the energy and ethanol intake in male survivors, the changes in many elements of a comprehensive range of nutrients and food groups were similar to those of controls, indicating that most dietary changes according to cancer diagnosis and subsequent survival were not significant.

A greater reduction in total energy intake in survivors was observed among men only. A previous study that included male survivors^11^ reported decreased energy intake, consistent with our results, although that study did not compare changes with those in cancer-free controls. In the case of females, a prospective study reported no difference in the reduction of energy intake over 6 years between survivors and controls^17^. These results suggest that changes in total energy intake over time for female survivors may not be particularly differ from changes in those without a cancer diagnosis.

We found that ethanol intake was decreased in male cancer survivors. Previous studies examining changes in ethanol intake are limited. Consistent with our findings, a study^11^ examining men and women combined found that intake was decreased in survivors, although there was no comparison with cancer-free controls, while another study^17^ in females showed no change among survivors and no difference compared with controls, again consistent with our findings. For women, the proportion of those who did not drink was > 60% of respondents for both questionnaires, so it is possible that alcohol-related intake changes could not be detected. Alcohol is considered to be a risk for several types of cancer^6^, and avoidance of alcohol is recommended to prevent new primary tumors in survivors^5^.

Among major site-specific cancer survivors, alcohol intake reduction in men with gastric cancer was the most prominent change from pre-diagnosis to subsequent survival in men. However, given that this study could not use information on cancer stage or treatment, it cannot be ruled out that the decrease may be attributable to gastric cancer-specific treatments rather than a change to a healthier lifestyle.

A slightly larger but insignificant increment was observed in vegetable, but not fruit, intakes for cancer survivors, especially for breast cancer survivors, than for cancer-free controls over five years among women only. Previous studies of breast cancer patients in Europe, which compared dietary changes prospectively with those for controls^17,18^, reported a slightly larger increment of intakes of both fruit and vegetables in survivors than in cancer-free participants (18.1 g and 13.6 g for the differences in changes of fruit and vegetable intake, respectively^17^, or 3.3% [equivalent to about 20 g] for total fruit and vegetables^18^) among a larger number of breast cancer cases(n = 563^17^ or 2699^18^), contrary to our findings. This difference in findings might be owing to the small number of breast cancer survivors (158 cases) caused by the relatively low incidence of this cancer in Japan^2^, although the reported difference in increments was at most 13 g or 3.3% between survivors and cancer-free controls^17,18^. Also, no differences were observed in the reductions in red meat intake between cancer survivors and controls in either gender. A previous study in women reported no difference in red meat intake reduction^17^, similar to our results. These results also suggest that dietary changes in intakes among survivors may not be attributed to a cancer diagnosis.

There are some limitations to this study. First, the period from diagnosis to the follow-up dietary survey was uneven. The mean interval was 2.1 years. One report noted that dietary changes were largest immediately after the cancer diagnosis (during the treatment/recovery period), after which intakes gradually returned to levels before diagnosis^31^. Thus, due to the short observation period, the results may have overestimated dietary changes as shown in separate calculations by observation period for energy and ethanol intake among men. In addition, survivors would have included severe cancers, which also would have led to an overestimation of changes. Second, rice, a traditional Japanese staple food, is generally consumed in a refined form. Although this study did not examine the consumption of whole grains, it is unlikely that many Japanese people consume unrefined brown rice frequently^32^, albeit that further research on this question is warranted.

Evidence to date on the diet of cancer survivors in relation to prognosis includes studies that use the diet prior to diagnosis as exposure^33–37^. Our findings of few and small changes from before the diagnosis in several nutrients and food groups in survivors indicate that cancer diagnosis and subsequent early survival do not cause drastic dietary changes. These findings may aid in the evaluation of the contribution of pre-diagnosis diets and may create future research opportunities on dietary habits in association with prognosis.

In conclusion, the dietary changes over five years, including before and after cancer diagnosis, in cancer survivors were small compared with changes among cancer-free controls in a prospective large-scale population-based cohort study. These findings suggest that most dietary changes in cancer survivors are not systematically different from those that occur in people without a cancer diagnosis.

## Supplementary Information

Supplementary Information.

## Data Availability

The minimal datasets used and/or analyzed during the current study available from Dr. Norie Sawada, Principal Investigator of the JPHC Study, on reasonable request. For information on how to submit an application for gaining access to JPHC data and/or biospecimens, please follow the instructions at https://epi.ncc.go.jp/en/jphc/805/8155.html.

## Abbreviations

BMI: Body mass index
CI: Confidence intervals
FFQ: Food frequency questionnaire
IQR: Interquartile range
JPHC Study: Japan public health center-based prospective study
PHC: Public health center
