# Supplementary material for: Prospective study of dietary changes in cancer survivors for five years including pre- and post- diagnosis compared with those in cancer-free participants
Source: Sci Rep. 2023 Jan 18;13:982. doi: 10.1038/s41598-023-27820-z (PMC9849447; doi:10.1038/s41598-023-27820-z)
Supplement: Supplementary file 1 — Supplementary Information. [file 41598_2023_27820_MOESM1_ESM.docx]

**Supplementary tables**

**Prospective study of dietary changes in cancer survivors for five years including pre- and post-diagnosis compared with those in cancer-free participants**

**Yuri Ishii^1^, Ribeka Takachi^2,5*^, Junko Ishihara^3,5^, Taiki Yamaji^4^, Motoki Iwasaki ^1,4^, Manami Inoue^1,5^, Shoichiro Tsugane^1,6^, Norie Sawada^1^**

Supplementary table S1. Changes in crude intakes of nutrients from baseline for male cancer survivors compared with male controls (n = 33,643)

|  | Cancer-free controls (n = 32757) | | | | | |  | All cancers (n = 886) | | | | | |  |  |  | Colorectal cancer (n = 226) | | | | | |  | Gastric cancer (n = 244) | | | | |  | Other cancers (n = 416) | | | | | | |  |  |
| --- | --- | --- | --- | --- | --- | --- | --- | --- | --- | --- | --- | --- | --- | --- | --- | --- | --- | --- | --- | --- | --- | --- | --- | --- | --- | --- | --- | --- | --- | --- | --- | --- | --- | --- | --- | --- | --- | --- |
|  | baseline median | Change from baseline | | | | |  | baseline median | Change from baseline | | | | |  | *P*^a^ |  | baseline median | Change from baseline | | | | |  | baseline median | Change from baseline | | | |  | baseline median | Change from baseline | | | | | |  | *P*^b^ |
|  |  | median |  | IQR | |  |  |  | median |  | IQR | |  |  |  |  |  | median |  | IQR | |  |  |  | median |  | IQR |  |  |  | median |  | IQR | | |  |  |  |
| Nutrients units/d |  |  |  | |  | |  |  |  |  |  |  |  |  |  |  |  |  |  | |  |  |  |  |  |  | | |  |  |  |  | |  |  |  |  |  |
| Protein (g) | 70.2 | −3.1 | (−20.2–14.5) | | | | * | 70.3 | −5.0 | (−24.7–14.8) | | | | * | .10 |  | 74.5 | −5.3 | (−24.0–15.6) | | | |  | 71.1 | −7.8 | (−27.8–10.4) | | | * | 67.9 | −3.7 | (−22.8–17.9) | | | | |  | .05 |
| Lipids (g) | 51.7 | −2.6 | (−19.2–14.2) | | | | * | 51.9 | −3.2 | (−18.9–14.6) | | | | * | .87 |  | 54.3 | −5.3 | (−22.7–14.5) | | | |  | 50.0 | −3.4 | (−17.7–11.9) | | |  | 51.7 | −1.7 | (−17.3–17.8) | | | | |  | .34 |
| SFA (g) | 15.33 | −1.41 | (−6.66–3.73) | | | | * | 15.39 | −1.49 | (−6.84–3.58) | | | | * | .99 |  | 16.11 | −2.25 | (−7.11–3.54) | | | | * | 14.77 | −1.33 | (−6.35–2.93) | | | * | 15.72 | −1.45 | (−6.32–4.73) | | | | |  | .54 |
| MUFA (g) | 17.86 | −0.39 | (−6.50–6.13) | | | | * | 17.66 | −0.39 | (−6.68–6.37) | | | |  | .91 |  | 18.63 | −1.16 | (−8.60–5.83) | | | |  | 16.94 | −0.98 | (−6.47–5.19) | | |  | 17.60 | −0.03 | (−5.68–7.31) | | | | |  | .23 |
| PUFA (g) | 11.81 | −0.43 | (−3.98–3.12) | | | | * | 11.70 | −0.67 | (−4.10–3.16) | | | |  | .66 |  | 12.46 | −1.24 | (−4.23–2.97) | | | |  | 11.32 | −0.70 | (−4.64–2.39) | | |  | 11.65 | −0.56 | (−3.62–4.06) | | | | |  | .22 |
| n-3 PUFA (g) | 2.28 | −0.20 | (−0.98–0.57) | | | | * | 2.27 | −0.21 | (−1.06–0.64) | | | | * | .88 |  | 2.49 | −0.25 | (−1.04–0.67) | | | |  | 2.27 | −0.23 | (−1.22–0.52) | | | * | 2.22 | −0.18 | (−1.03–0.69) | | | | |  | .61 |
| n-6 PUFA (g) | 9.41 | −0.25 | (−3.03–2.60) | | | | * | 9.38 | −0.44 | (−3.30–2.62) | | | |  | .53 |  | 9.86 | −0.98 | (−3.75–2.47) | | | |  | 8.96 | −0.34 | (−3.36–2.15) | | |  | 9.23 | −0.07 | (−3.11–3.25) | | | | |  | .16 |
| Cholesterol (mg) | 279 | −35 | (−129–57) | | | | * | 294 | −33 | (−140–60) | | | | * | .55 |  | 297 | −39 | (−143–52) | | | | * | 296 | −31 | (−127–55) | | | * | 287 | −30 | (−146–67) | | | | | * | .88 |
| Carbohydrate (g) | 273.4 | 3.8 | (−50.5–54.5) | | | | * | 271.9 | −4.3 | (−65.0–47.0) | | | |  | **< .01** |  | 276.9 | 4.5 | (−52.3–52.8) | | | |  | 285.5 | −21.9 | (−86.4–34.9) | | | * | 261.0 | 1.0 | (−58.8–53.3) | | | | |  | **< .001** |
| Total dietary fiber (g) † | 11.1 | 0.7 | (−2.7–4.3) | | | | * | 11.7 | 0.4 | (−3.1–5.2) | | | | * | .88 |  | 12.1 | 0.5 | (−2.7–5.8) | | | | * | 11.7 | −0.1 | (−3.5–4.6) | | |  | 11.4 | 0.9 | (−3.0–5.3) | | | | | * | .50 |
| Soluble dietary fiber (g) † | 2.5 | 0.1 | (−0.7–1.1) | | | | * | 2.7 | 0.2 | (−0.8–1.3) | | | | * | .33 |  | 2.7 | 0.2 | (−0.9–1.4) | | | |  | 2.6 | 0.1 | (−0.9–1.2) | | |  | 2.7 | 0.2 | (−0.7–1.3) | | | | | * | .56 |
| Insoluble dietary fiber (g) † | 7.9 | 0.6 | (−1.7–3.1) | | | | * | 8.2 | 0.5 | (−2.0–3.6) | | | | * | .90 |  | 8.5 | 0.7 | (−1.8–4.0) | | | | * | 8.4 | 0.1 | (−2.7–3.2) | | |  | 8.0 | 0.7 | (−2.0–3.6) | | | | | * | .32 |
| Potassium (mg) | 2578 | −56 | (-736–627) | | | | * | 2684 | −65 | (−771–753) | | | |  | .87 |  | 2796 | −54 | (−859–712) | | | |  | 2731 | −173 | (−809–611) | | |  | 2585 | −18 | (−730–838) | | | | |  | .42 |
| Calcium (mg) | 456 | 0 | (−140–144) | | | |  | 469 | 5 | (−130–183) | | | |  | .14 |  | 471 | 16 | (−128–201) | | | |  | 475 | −16 | (−179–163) | | |  | 465 | 16 | (−114–186) | | | | |  | .13 |
| Magnesium (mg) | 311 | −1 | (−77–74) | | | |  | 313 | −15 | (−88–77) | | | |  | .10 |  | 328 | −18 | (−86–77) | | | |  | 314 | −24 | (−110–54) | | | * | 298 | −2 | (−77–89) | | | | |  | .03 |
| Phosphorus (mg) | 1080 | −41 | (−299–216) | | | | * | 1088 | −80 | (−332–246) | | | | * | .14 |  | 1120 | −74 | (−314–263) | | | |  | 1118 | −116 | (−393–185) | | | * | 1042 | −70 | (−310–282) | | | | |  | .09 |
| Iron (mg) | 8.8 | 0.0 | (−2.1–2.0) | | | |  | 9.1 | −0.2 | (−2.4–2.2) | | | |  | .16 |  | 9.3 | −0.4 | (−2.6–2.2) | | | |  | 9.2 | −0.6 | (−2.9–1.5) | | | * | 8.9 | 0.0 | (−2.0–2.6) | | | | |  | .04 |
| Zinc (mg) | 8.4 | −0.1 | (−2.0–1.7) | | | | * | 8.5 | −0.4 | (−2.3–1.8) | | | | * | .03 |  | 8.8 | −0.4 | (−2.2–1.8) | | | |  | 8.7 | −0.8 | (−2.7–1.1) | | | * | 8.3 | −0.2 | (−2.2–2.2) | | | | |  | **< .01** |
| Copper (mg) | 1.25 | 0.01 | (−0.25–0.28) | | | | * | 1.26 | −0.02 | (−0.28–0.27) | | | |  | .04 |  | 1.30 | −0.02 | (−0.27–0.24) | | | |  | 1.31 | −0.11 | (−0.33–0.19) | | | * | 1.21 | 0.02 | (−0.26–0.33) | | | | |  | **< .001** |
| Manganese (mg) | 4.06 | 0.03 | (−0.95–0.98) | | | |  | 4.18 | −0.26 | (−1.38–0.94) | | | | * | **< .001** |  | 4.24 | −0.06 | (−1.37–0.94) | | | |  | 4.34 | −0.62 | (−1.59–0.66) | | | * | 4.05 | −0.09 | (−1.19–1.09) | | | | |  | **< .001** |
| Retinol (μg) | 423 | −92 | (−410–51) | | | | * | 420 | −95 | (−400–62) | | | | * | .78 |  | 456 | −133 | (−475–63) | | | | * | 407 | −80 | (−359–53) | | | * | 406 | −95 | (−396–69) | | | | | * | .64 |
| Retinol eq (μg) | 738 | −135 | (−493–139) | | | | * | 759 | −112 | (−500–197) | | | | * | .17 |  | 794 | −134 | (−593–196) | | | | * | 734 | −87 | (−472–220) | | | * | 748 | −109 | (−440–180) | | | | | * | .33 |
| α−Carotene (μg) | 381 | −9 | (−303–217) | | | | * | 399 | −4 | (−291–271) | | | |  | .22 |  | 413 | −2 | (−263–245) | | | |  | 344 | −13 | (−322–253) | | |  | 411 | −1 | (−288–289) | | | | |  | .60 |
| β−Carotene (μg) | 2447 | −99 | (−1196–964) | | | | * | 2697 | −57 | (−1216–1248) | | | |  | .21 |  | 2728 | −46 | (−1017–1175) | | | |  | 2598 | −22 | (−1410–1331) | | |  | 2721 | −84 | (−1290–1188) | | | | |  | .65 |
| Vitamin D (μg) | 9.5 | −1.8 | (−6.0–1.9) | | | | * | 9.7 | −1.9 | (−6.6–2.5) | | | | * | .49 |  | 10.5 | −1.9 | (−6.7–1.8) | | | | * | 10.3 | −2.0 | (−7.0–2.7) | | | * | 9.0 | −1.9 | (−6.2–2.5) | | | | | * | .87 |
| Vitamin K (μg) | 198 | 6 | (−71–93) | | | | * | 212 | 5 | (−81–108) | | | |  | .91 |  | 220 | 4 | (−87–112) | | | |  | 217 | 1 | (−95–100) | | |  | 206 | 8 | (−70–108) | | | | |  | .59 |
| Vitamin B1 (mg) | 0.96 | −0.06 | (−0.35–0.21) | | | | * | 0.97 | −0.06 | (−0.35–0.23) | | | | * | .95 |  | 1.03 | −0.09 | (−0.38–0.20) | | | | * | 0.99 | −0.10 | (−0.40–0.14) | | | * | 0.92 | −0.01 | (−0.30–0.30) | | | | |  | .02 |
| Vitamin B2 (mg) | 1.33 | −0.08 | (−0.45–0.27) | | | | * | 1.40 | −0.09 | (−0.48–0.29) | | | | * | .56 |  | 1.45 | −0.12 | (−0.54–0.27) | | | | * | 1.43 | −0.12 | (−0.55–0.23) | | | * | 1.38 | −0.07 | (−0.41–0.32) | | | | |  | .33 |
| Niacin (mg) | 19.4 | −1.0 | (−6.2–4.3) | | | | * | 18.9 | −1.7 | (−7.6–4.0) | | | | * | **< .01** |  | 20.0 | −2.2 | (−8.0–3.9) | | | | * | 19.5 | −2.5 | (−8.5–3.4) | | | * | 17.9 | −1.2 | (−6.4–4.6) | | | | |  | **< .01** |
| Vitamin B6 (mg) | 1.5 | 0.0 | (−0.4–0.3) | | | | * | 1.5 | −0.1 | (−0.5–0.4) | | | | * | .03 |  | 1.6 | −0.1 | (−0.5–0.4) | | | |  | 1.6 | −0.1 | (−0.6–0.2) | | | * | 1.5 | 0.0 | (−0.4–0.4) | | | | |  | .02 |
| Vitamin B12 (μg) | 8.6 | −1.5 | (−5.0–1.6) | | | | * | 8.8 | −1.8 | (−5.9–1.8) | | | | * | .22 |  | 9.7 | −1.6 | (−5.8–1.7) | | | | * | 9.3 | −2.2 | (−7.3–1.2) | | | * | 8.2 | −1.5 | (−5.3–2.3) | | | | | * | .14 |
| Vitamin C (mg) | 106 | −4 | (−43–36) | | | | * | 119 | −1 | (−49–44) | | | |  | .38 |  | 122 | 2 | (−57–46) | | | |  | 122 | −9 | (−49–43) | | |  | 117 | 0 | (−46–43) | | | | |  | .53 |

IQR, Interquartile range; SFA, Saturated fatty acid; MUFA, Monounsaturated fatty acids; PUFA, Polyunsaturated fatty acids. * Statistical significance was set at P < .01 and the Wilcoxon signed−rank test was used to determine the significance of differences in intake between the baseline and follow-up survey. ^a^ The Mann–Whitney *U*−test was used for the difference in change between cancer-free male controls and survivors of all cancers, ^b^ The Kruskal−Wallis test was used for comparison of the change among cancer-free male controls and survivors of specific cancer types. Statistical significance was set at *P* < .01 for both test. †Dietary fiber was measured by the modified Prosky method based on the AOAC (AOAC 991.42 and AOAC 993.19) method [28]. Significant values are in [bold].

Supplementary table S2. Changes in crude intakes of nutrients from baseline for female cancer survivors compared with female controls (n = 39,549)

|  | Cancer-free controls (n = 38903) | | |  |  | All cancers (n = 646) | | | | |  | Colorectal cancer (n = 134) | | |  |  | Gastric cancer (n = 91) | | |  |  | Breast cancer (n = 158) | | |  |  | Other cancers (n = 263) | | | | |
| --- | --- | --- | --- | --- | --- | --- | --- | --- | --- | --- | --- | --- | --- | --- | --- | --- | --- | --- | --- | --- | --- | --- | --- | --- | --- | --- | --- | --- | --- | --- | --- |
|  | baseline median | Change from baseline | | |  | baseline median | Change from baseline | |  | *P*^a^ |  | baseline median | Change from baseline | | |  | baseline median | Change from baseline | | |  | baseline median | Change from baseline | | |  | baseline median | Change from baseline | |  | *P*^b^ |
|  |  | median | IQR | |  |  | median | IQR |  |  |  |  | median | IQR | |  |  | median | IQR | |  |  | median | IQR | |  |  | median | IQR |  |  |
| Nutrients units/d |  |  |  | |  |  |  |  |  |  |  |  |  |  | |  |  |  |  | |  |  |  |  | |  |  |  |  |  |  |
| Protein (g) | 67.5 | −2.7 | (−18.8–13.8) | | * | 66.9 | −3.6 | (−19.1–14.2) |  | .81 |  | 66.4 | −2.1 | (−13.2–20.0) | |  | 71.3 | −10.6 | (−27.8–5.6) | | * | 65.6 | −1.0 | (−16.0–15.6) | |  | 66.3 | −4.3 | (−20.9–13.5) |  | .03 |
| Lipid (g) | 53.7 | −2.1 | (−17.7–14.1) | | * | 53.0 | −1.8 | (−17.2–12.9) |  | .93 |  | 53.2 | −0.5 | (−16.2–14.6) | |  | 56.5 | −5.3 | (−21.8–10.5) | |  | 53.4 | −0.1 | (−14.6–14.5) | |  | 51.6 | −3.1 | (−19.6–11.4) |  | .31 |
| SFA (g) | 16.13 | −1.35 | (−6.53–3.67) | | * | 15.88 | −1.57 | (−6.77–3.29) | * | .81 |  | 15.61 | −1.18 | (−5.54–4.26) | |  | 15.80 | −1.77 | (−6.86–2.65) | |  | 16.73 | −0.96 | (−5.49–3.95) | |  | 15.76 | −2.20 | (−7.76–2.94) | * | .35 |
| MUFA (g) | 18.29 | −0.04 | (−5.76–6.22) | | * | 18.16 | −0.29 | (−5.73–5.77) |  | .89 |  | 18.30 | 0.60 | (−4.56–5.52) | |  | 19.07 | −1.97 | (−6.43–4.36) | |  | 18.51 | 0.77 | (−5.22–7.83) | |  | 17.90 | −0.58 | (−6.21–5.64) |  | .36 |
| PUFA (g) | 12.18 | −0.36 | (−3.75–3.17) | | * | 12.04 | −0.37 | (−3.45–3.15) |  | .89 |  | 12.18 | −0.04 | (−2.52–2.52) | |  | 12.55 | −1.16 | (−4.95–1.42) | |  | 11.80 | 0.24 | (−3.44–3.97) | |  | 11.87 | −0.71 | (−3.56–3.16) |  | .18 |
| n−3 PUFA (g) | 2.36 | −0.18 | (−0.92–0.57) | | * | 2.42 | −0.19 | (−0.83–0.60) | * | .61 |  | 2.44 | −0.11 | (−0.63–0.75) | |  | 2.73 | −0.42 | (−1.36–0.25) | | * | 2.38 | −0.20 | (−0.74–0.59) | |  | 2.27 | −0.14 | (−0.84–0.61) |  | .04 |
| n−6 PUFA (g) | 9.72 | −0.20 | (−2.89–2.66) | | * | 9.61 | −0.26 | (−2.79–2.58) |  | .97 |  | 9.71 | −0.05 | (−2.23–2.12) | |  | 9.87 | −1.04 | (−3.34–1.54) | |  | 9.64 | 0.45 | (−2.84–3.22) | |  | 9.48 | −0.36 | (−2.86–2.52) |  | .30 |
| Cholesterol (mg) | 268 | −31 | (−118–52) | | * | 254 | −31 | (−107–57) | * | .45 |  | 265 | −24 | (−95–61) | |  | 286 | −41 | (−156–26) | | * | 244 | −14 | (−86–57) | |  | 248 | −36 | (−115–61) | * | .11 |
| Carbohydrate (g) | 250.2 | −10.2 | (−55.7–33.7) | | * | 249.4 | −11.5 | (−61.0–31.2) | * | .55 |  | 249.9 | −1.7 | (−56.4–41.5) | |  | 264.3 | −36.4 | (−79.5–26.3) | |  | 251.6 | −3.3 | (−55.8–34.1) | |  | 244.7 | −16.2 | (−60.5–25.2) | * | .11 |
| Total dietary fiber (g) † | 13.1 | 0.9 | (−2.7–4.9) | | * | 13.4 | 1.4 | (−2.8–5.4) | * | .32 |  | 13.5 | 1.5 | (−2.2–5.5) | | * | 14.3 | 0.9 | (−3.9–5.3) | |  | 13.2 | 1.9 | (−2.3–6.2) | | * | 12.9 | 1.2 | (−3.0–5.0) |  | .31 |
| Soluble dietary fiber (g) † | 3.1 | 0.2 | (−0.8–1.3) | | * | 3.2 | 0.3 | (−0.8–1.3) | * | .19 |  | 3.1 | 0.5 | (−0.6–1.5) | | * | 3.6 | 0.1 | (−0.9–1.6) | |  | 3.1 | 0.5 | (−0.8–1.5) | | * | 3.0 | 0.2 | (−0.9–1.3) |  | .23 |
| Insoluble dietary fiber(g) † | 9.2 | 0.7 | (−1.8–3.5) | | * | 9.4 | 1.0 | (−1.7–3.7) | * | .20 |  | 9.6 | 0.7 | (−1.4–4.1) | | * | 10.3 | 0.4 | (−2.4–3.7) | |  | 9.0 | 1.5 | (−1.4–4.1) | | * | 9.2 | 1.0 | (−2.0–3.6) | * | .31 |
| Potassium (mg) | 2776 | −8 | (−690–707) | |  | 2735 | 48 | (−616–793) |  | .18 |  | 2759 | 234 | (−522–827) | |  | 2965 | −232 | (−774–567) | |  | 2710 | 187 | (−518–1067) | |  | 2619 | −17 | (−742–704) |  | .01 |
| Calcium (mg) | 514 | 16 | (−136–174) | | * | 529 | 19 | (−150–181) |  | .98 |  | 546 | 61 | (−118–229) | |  | 576 | −69 | (−207–178) | |  | 513 | 45 | (−60–214) | | * | 522 | 0 | (−178–140) |  | **< .01** |
| Magnesium (mg) | 309 | 1 | (−71–77) | | * | 312 | 0 | (−73–73) |  | .67 |  | 307 | 18 | (−63–90) | |  | 344 | −33 | (−134–43) | |  | 312 | 23 | (−44–86) | |  | 305 | −9 | (−84–68) |  | **< .01** |
| Phosphorus (mg) | 1054 | −35 | (−280–211) | | * | 1054 | −46 | (−291–228) |  | .89 |  | 1060 | 16 | (−245–255) | |  | 1153 | −194 | (−383–165) | | * | 1026 | 5 | (−207–262) | |  | 1041 | −62 | (−310–215) |  | .01 |
| Iron (mg) | 8.8 | 0.0 | (−1.9–2.1) | | * | 8.8 | 0.0 | (−1.8–2.2) |  | .61 |  | 9.1 | 0.4 | (−1.3–2.4) | |  | 9.4 | −0.6 | (−3.0–1.7) | |  | 8.5 | 0.7 | (−1.4–2.3) | |  | 8.7 | −0.1 | (−2.1–2.0) |  | .02 |
| Zinc (mg) | 7.9 | −0.3 | (−1.9–1.4) | | * | 7.8 | −0.3 | (−2.0–1.4) |  | .69 |  | 7.9 | −0.2 | (−1.5–2.0) | |  | 8.2 | −1.4 | (−2.7–0.9) | | * | 7.6 | 0.1 | (−1.6–1.6) | |  | 7.8 | −0.5 | (−2.1–1.2) |  | **< .01** |
| Copper (mg) | 1.22 | −0.01 | (−0.25–0.25) | |  | 1.24 | 0.00 | (−0.25–0.25) |  | .95 |  | 1.22 | 0.02 | (−0.19–0.28) | |  | 1.37 | −0.11 | (−0.41–0.20) | |  | 1.23 | 0.06 | (−0.20–0.31) | |  | 1.22 | −0.01 | (−0.27–0.21) |  | .03 |
| Manganese (mg) | 3.94 | −0.03 | (−0.99–0.90) | | * | 3.97 | −0.03 | (−0.98–0.95) |  | .81 |  | 3.95 | 0.36 | (−0.73–1.04) | |  | 4.01 | −0.27 | (−1.37–0.79) | |  | 3.78 | 0.07 | (−0.68–1.04) | |  | 4.02 | −0.18 | (−1.15–0.77) |  | .06 |
| Retinol (μg) | 359 | −67 | (−349–46) | | * | 329 | −72 | (−341–51) | * | .95 |  | 276 | −46 | (−306–76) | | * | 310 | −51 | (−351–120) | |  | 402 | −102 | (−341–59) | | * | 325 | −79 | (−358–24) | * | .83 |
| Retinol eq (μg) | 781 | −113 | (−442–154) | | * | 794 | −85 | (−452–202) | * | .43 |  | 761 | −65 | (−366–191) | |  | 863 | −35 | (−637–226) | |  | 825 | −56 | (−440–281) | |  | 743 | −115 | (−457–141) | * | .65 |
| α−Carotene (μg) | 630 | −8 | (−345–296) | | * | 628 | 2 | (−332–327) |  | .08 |  | 639 | 5 | (−217–339) | |  | 621 | 2 | (−428–367) | |  | 639 | 9 | (−289–334) | |  | 621 | −15 | (−363–277) |  | .24 |
| β−Carotene (μg) | 3322 | −94 | (−1365–1186) | | * | 3244 | 59 | (−1266–1338) |  | .06 |  | 3265 | 9 | (−916–1379) | |  | 3534 | 274 | (−1698–1709) | |  | 3199 | 148 | (−1035–1567) | |  | 3134 | −94 | (−1440–1073) |  | .15 |
| Vitamin D (μg) | 9.8 | −1.6 | (−5.7–2.0) | | * | 9.6 | −1.2 | (−5.1–2.3) | * | .10 |  | 9.9 | −1.3 | (−4.7–2.5) | |  | 10.1 | −2.8 | (−6.7–0.6) | | * | 9.6 | −1.1 | (−5.1–2.4) | |  | 9.4 | −1.0 | (−4.8–2.3) | * | .06 |
| Vitamin K (μg) | 235 | 13 | (−70–111) | | * | 243 | 20 | (−67–127) | * | .40 |  | 242 | 21 | (−59–151) | |  | 292 | 4 | (−114–118) | |  | 246 | 38 | (−68–163) | | * | 234 | 17 | (−57–111) |  | .34 |
| Vitamin B1 (mg) | 1.00 | −0.06 | (−0.34–0.21) | | * | 0.99 | −0.07 | (−0.33–0.20) | * | .51 |  | 0.96 | 0.00 | (−0.28–0.24) | |  | 1.09 | −0.15 | (−0.47–0.08) | | * | 1.01 | −0.02 | (−0.31–0.24) | |  | 0.97 | −0.09 | (−0.33–0.18) | * | .06 |
| Vitamin B2 (mg) | 1.38 | −0.04 | (−0.41–0.32) | | * | 1.41 | −0.03 | (−0.41–0.34) |  | .42 |  | 1.39 | 0.11 | (−0.28–0.40) | |  | 1.53 | −0.08 | (−0.46–0.17) | |  | 1.38 | 0.03 | (−0.34–0.34) | |  | 1.41 | −0.08 | (−0.49–0.36) |  | .04 |
| Niacin (mg) | 17.6 | −0.7 | (−5.2–4.0) | | * | 17.0 | −0.4 | (−5.1–4.1) |  | .63 |  | 17.1 | −0.3 | (−3.9–4.3) | |  | 17.8 | −2.7 | (−7.7–2.0) | | * | 17.1 | 0.1 | (−4.7–4.7) | |  | 16.7 | −0.3 | (−5.2–4.4) |  | .06 |
| Vitamin B6 (mg) | 1.4 | 0.0 | (−0.4–0.3) | | * | 1.4 | 0.0 | (−0.3–0.4) |  | .10 |  | 1.4 | 0.1 | (−0.3–0.4) | |  | 1.5 | −0.1 | (−0.5–0.3) | |  | 1.4 | 0.1 | (−0.3–0.4) | |  | 1.4 | 0.0 | (−0.3–0.3) |  | .03 |
| Vitamin B12 (μg) | 8.3 | −1.3 | (−4.4–1.7) | | * | 8.2 | −1.1 | (−4.1–1.3) | * | .73 |  | 8.2 | −1.1 | (−3.8–1.0) | | * | 9.2 | −2.5 | (−6.4–0.5) | | * | 8.0 | −1.0 | (−3.5–1.7) | | * | 7.8 | −1.0 | (−3.9–1.6) | * | .12 |
| Vitamin C (mg) | 140 | −2 | (−48–46) | | * | 146 | 0 | (−48–54) |  | .24 |  | 141 | 10 | (−32–55) | |  | 159 | −10 | (−58–62) | |  | 148 | 5 | (−46–60) | |  | 145 | −6 | (−58–47) |  | .20 |

IQR, Interquartile range; SFA, Saturated fatty acid; MUFA, Monounsaturated fatty acids; PUFA, Polyunsaturated fatty acids. * Statistical significance was set at P < .01 and The Wilcoxon signed−rank test was used to determine the significance of differences in intake between the baseline and follow-up survey. ^a^ The Mann–Whitney *U*−test was used for the difference in change between cancer-free female controls and survivors of all cancers, ^b^ The Kruskal−Wallis test was used for comparison of the change among cancer-free female controls and survivors of specific cancer types. Statistical significance was set at *P* < .01 for both test. †Dietary fiber was measured by the modified Prosky method based on the AOAC (AOAC 991.42 and AOAC 993.19) method [28]. Significant values are in [bold].
